# Glucose variability and the risks of stroke, myocardial infarction, and all-cause mortality in individuals with diabetes: retrospective cohort study

**DOI:** 10.1186/s12933-020-01134-0

**Published:** 2020-09-22

**Authors:** Da Young Lee, Kyungdo Han, Sanghyun Park, Ji Hee Yu, Ji A. Seo, Nam Hoon Kim, Hye Jin Yoo, Sin Gon Kim, Kyung Mook Choi, Sei Hyun Baik, Yong Gyu Park, Nan Hee Kim

**Affiliations:** 1 Division of Endocrinology and Metabolism, Department of Internal Medicine, Korea University College of Medicine, Seoul, Republic of Korea; 2 Department of Biostatics, College of Medicine, The Catholic University of Korea, 222, Banpo-daero, Seocho-gu, Seoul, 06591 Republic of Korea; 3 Division of Endocrinology and Metabolism, Department of Internal Medicine, Korea University Ansan Hospital, Korea University College of Medicine, Danwon-gu, Ansan-si, Gyeonggi-do 15355 Republic of Korea

**Keywords:** Diabetes mellitus Glucose variability, Cardiovascular disease, Stroke, All-cause mortality, The Korean National Health Insurance Corporation

## Abstract

**Background:**

Previous research regarding long-term glucose variability over several years which is an emerging indicator of glycemic control in diabetes showed several limitations. We investigated whether variability in long-term fasting plasma glucose (FG) can predict the development of stroke, myocardial infarction (MI), and all-cause mortality in patients with diabetes.

**Methods:**

This is a retrospective cohort study using the data provided by the Korean National Health Insurance Corporation. A total of 624,237 Koreans ≥ 20 years old with diabetes who had undergone health examinations at least twice from 2005 to 2008 and simultaneously more than once from 2009 to 2010 (baseline) without previous histories of stroke or MI. As a parameter of variability of FG, variability independent of mean (VIM) was calculated using FG levels measured at least three times during the 5 years until the baseline. Study endpoints were incident stroke, MI, and all-cause mortality through December 31, 2017.

**Results:**

During follow-up, 25,038 cases of stroke, 15,832 cases of MI, and 44,716 deaths were identified. As the quartile of FG VIM increased, the risk of clinical outcomes serially increased after adjustment for confounding factors including duration and medications of diabetes and the mean FG. Adjusted hazard ratios (95% confidence intervals) of FG VIM quartile 4 compared with quartile 1 were 1.20 (1.16–1.24), 1.20 (1.15–1.25), and 1.32 (1.29–1.36) for stroke, MI and all-cause mortality, respectively. The impact of FG variability was higher in the elderly and those with a longer duration of diabetes and lower FG levels.

**Conclusions:**

In diabetes, long-term glucose variability showed a dose–response relationship with the risk of stroke, MI, and all-cause mortality in this nationwide observational study.

## Background

For individuals with diabetes, maintaining optimal blood glucose level is required to prevent complications and death [1]. However, as incident cardiovascular disease (CVD) and deaths associated with diabetes cannot be fully explained by increased HbA1c itself [2–4], more attention has been focused on non-traditional risk factors such as glucose variability (GV). Patients with diabetes with similar HbA1c values can have different daily glycemic profiles with variations in the duration and frequency of glycemic excursions [5]. Short-term GV usually means same-day or between-day glycemic oscillations and might be influenced by the individuals’ diet, exercise, and treatment modality. Long-term GV represents the visit-to-visit variability in fasting glucose (FG) estimated over months to years; compliance with medication; and deterioration of insulin secretion and resistance, which can be important issues [6].

During the past decade, detrimental effects of GV on patients with diabetes have been proposed for various medical conditions [7–13]. GV could not only predict diabetic vascular complications [7, 14, 15], heart failure [9], and postoperative complications of aortic valve implantation [11], but also indicate poor prognosis for in-patients with acute lung diseases [8] and acute coronary syndrome [13]. Moreover, because GV could modify the correlation between time in range and estimated HbA1c, GV consideration was recommended when setting individualized goals for glycemic control [10].

Although there have been a few attempts to verify if GV can be a contributor to CVD or mortality [6, 7, 15–21], there is no consensus for patients with diabetes. Previous studies have yielded partial significance according to age, study group, or status of glycemic control [6, 7, 15–21]. In addition, they have several limitations such as small sample size and short-term follow-up periods.

In the present study, we used a nationwide population-based database in Korea to investigate whether long-term variability in FG can predict the risk of stroke, myocardial infarction (MI), and all-cause mortality during a median 8 years of follow-up, to specify the population prone to the risk of higher GV, and to compare it with single measurements of FG.

## Methods

### Study design and subjects

**Fig. 1:**
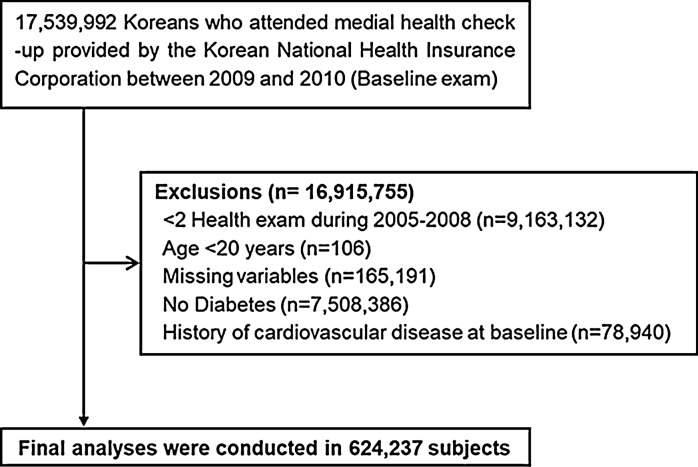
Selection of study subjects

As shown in Fig. 1, we analyzed the data of patients with diabetes who had undergone health examinations provided by the National Health Insurance Corporation (NHIC) at least twice from 2005 to 2008 and simultaneously more than once between January 1, 2009, and December 31, 2010 (baseline exam). Among them, we excluded individuals aged < 20 years and those with histories of stroke or MI, along with those missing data in the inclusion criteria. After those exclusions, 624,237 individuals were included in this study.

The NHIC is the single health insurance system operated by the Korean government and covers about 97% of the Korean population. It has an eligibility profile, health examination database (general health examinations and questionnaires on lifestyle), medical treatment information identified by the medical bills submitted by healthcare providers, and a medical care institution database [22, 23]. Enrollees are advised to undergo a standardized medical examination annually or biannually. The entire database of the NHIC is now open to all researchers.

The presence of diabetes was defined as at least one claim per year for the prescription of anti-diabetic medications under the International Classification of Diseases, Tenth Revision (ICD-10) codes E10–14 or a fasting plasma glucose level ≥ 7 mm ol/L. Type 1 diabetes (T1DM) was defined as ICD-10 code E10 with at least one prescription history of insulin. The remained subjects were referred to as type 2 diabetes (T2DM).

This study was approved by the official review committee in the NHIC and the Institutional Review Board of the Korea University Ansan Hospital (Institutional Review Board number 2019AS0158) and was carried out in accordance with the Helsinki Declaration of 1975.

### Definition of GV

**Fig. 2:**
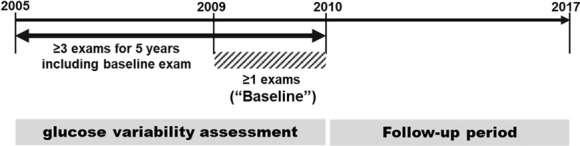
Assessment of glucose variability and follow-up period

The variability independent of the mean (VIM) of FG was used as a primary variability parameter calculated from FG levels measured at least three times during the 5 years prior to the baseline (Fig. 2). The FG level at baseline was included in calculation of FG variability. Additionally, standard deviation (SD), coefficient of variation (CV, SD/mean), and average real variability (ARV) of FG during the same period were calculated using the following equation:

$${\text{VIM}} = 100 \times \frac{\text{SD}}{{{\text{mean}}^{\beta } }},$$

where β is derived by nonlinear regression analysis, based on the natural logarithm of SD over the natural logarithm of the mean [24].

$${\text{ARV}} = \frac{1}{{{\text{N}} - 1}}\sum\nolimits_{{{\text{k}} = 1}}^{{{\text{n}} - 1}} { \times \left| {{\text{BP}}_{{{\text{K}} + 1}} - {\text{BP}}_{\text{K}} } \right|,}$$

where k ranges from 1 to n-1, and n is the number of FG measurements.

### Study outcomes

Incidences of stroke, MI, or death until December 31, 2017, were the endpoints of this study. Stroke was defined as the recording of ICD-10 codes I63 or I64 during admission with simultaneous claims for brain CT or MRI. MI was diagnosed by the recording of ICD-10 codes I21 or I22 with hospitalization. Deceased cases were identified based on nationwide death certificate data from the Korea National Statistical Office regardless of a previous diagnosis of stroke or MI. The time interval between the baseline examination and the date of study outcome or December 31, 2017 is defined as the follow-up period.

### Anthropometric and laboratory measurements

We used data from questionnaires regarding demographic characteristics, lifestyle, medical history, and medications during the medical examination. Alcohol consumption habit was divided into near abstinence, moderate (< 30 g/day), or severe (≥ 30 g/day). Smoking history was categorized as never, ex-, and current smokers. Doing regular exercise was defined > 20 min of vigorous-intensity or > 30 min of moderate-intensity exercise at least once per week [25]. Body mass index (BMI) was estimated by weight in kilograms divided by the square of height in meters. Waist circumference was checked at the middle point between the iliac crest and the rib cage.

The presence of hypertension was defined as systolic blood pressure (BP) ≥ 140 mmHg, diastolic BP ≥ 90 mmHg, or the presence of at least one prescription of anti-hypertensive medications under ICD-10 codes I10–I15 per year. The presence of malignancy was determined by registration in the Korea Central Cancer Registry under the International Classification of Diseases, ICD-10 C00-C96 before baseline examination. Low-income status was defined by the lowest 20%.

Venous blood samples were drawn in the morning after an overnight fast of at least 8 hours to measure the levels of plasma glucose, total cholesterol, triglycerides, high-density lipoprotein cholesterol, low-density lipoprotein cholesterol (LDL-C), and creatinine.

Dyslipidemia was defined as total cholesterol level ≥ 6.21 mmol/L or the presence of at least one claim per year for the prescription of anti-hyperlipidemic drugs under ICD-10 code E78. The estimated glomerular filtration rate (eGFR) was estimated by the Modification of Diet in Renal Disease formula [26], and eGFR < 60 mL/min/1.73 m^2^ was classified as chronic kidney disease (CKD) [27]. The number of oral anti-diabetic medication among metformin, sulfonylurea, meglitinide, thiazolidinedione, inhibitors of dipeptidyl peptidase 4 (DPP-4 inhibitors), and acarbose taken in the 12 months prior to baseline was identified. The history of heart disease was identified by self-report.

Quality control of the laboratory tests was conducted in accordance with the Korean Association of Laboratory Quality Control.

### Statistical analysis

Data are presented as mean ± SD, geometric mean (95% confidence intervals [CIs]), or number (%). The baseline characteristics were compared using Chi square tests for categorical variables and analysis of variance for continuous variables after dividing the subjects according to the FG VIM quartile. Triglyceride levels were log-transformed for analysis.

To assess the risks of stroke, MI, and all-cause mortality, we conducted multivariate-adjusted Cox proportional hazards analyses according to FG VIM quartiles and deciles, using quartile 1 or decile 1 as the reference group.

We adjusted for confounders at baseline using two models. Model 1 was adjusted for age, sex, BMI, alcohol drinking, smoking, regular exercise, presence of hypertension and dyslipidemia, CKD, and low-income status. Model 2 is the same as model 1, plus further adjustment for duration of diabetes over 5 years, the number of classes of oral anti-diabetic medication, insulin prescription history taken in the 12 months prior to baseline, and mean FG during the 5 years preceding the baseline exam.

We conducted several subgroup analyses to evaluate the effects of age; sex; BMI; smoking; income status; presence of hypertension, dyslipidemia, and malignancy; duration of diabetes; baseline FG level; subtype of diabetes; and anti-diabetic medication. The hazard ratios (HRs) and 95% CIs for stroke, MI, and all-cause death in FG VIM quartile 4 versus quartiles 1–3, with adjustments for confounders, were estimated.

To compare with the predictive value of a single FG level, we performed the above-mentioned Cox analysis according to baseline FG level, with FG levels of 100-119 mg/dL being the reference group. The mean of FG was excluded as a confounder in this analysis. Additionally, we repeated this analysis after stratifying the subjects according to prescription history of anti-diabetic medication.

We checked a variable inflation factor for all covariates of less than 2.0 and found no relevant multicollinearity in covariates. SAS version 9.3 (SAS Institute Inc., Cary, NC, USA) was used for statistical analysis. A *p* value of < 0.05 was considered to be statistically significant.

## Results

FG VIM quartile 4 had more males and current smokers and showed higher FG and triglyceride levels compared with quartile 1 (Table 1). On the other hand, this group was younger, had a lower BP and LDL-C level, and had lower proportions of hypertension, dyslipidemia, and heart disease versus quartile 1. About 46% of the highest VIM quartile was not treated with any anti-hyperglycemic agents. However, a higher proportion of them was treated with insulin for the preceding 12 months than other tertiles.

**Table 1:** Baseline characteristics of the study subjects according to quartiles of fasting glucose variability

Characteristics	VIM Q1 (n = 156061)	VIM Q2 (n = 156058)	VIM Q3 (n = 156060)	VIM Q4 (n = 156058)	*P* value
Age (years)	58.6 ± 10.6	57.4 ± 11.0	56.5 ± 11.7	54.7 ± 13.1	< 0.001
Sex, male (%)	98563 (63.2)	101947 (65.3)	103675 (66.4)	106869 (68.5)	< 0.001
BMI (kg/m^2^)	24.9 ± 3.0	25.0 ± 3.1	25.1 ± 3.2	24.9 ± 3.4	< 0.001
WC (cm)	85.2 ± 8.0	85.5 ± 8.0	85.6 ± 8.2	85.1 ± 8.6	< 0.001
Systolic BP (mmHg)	128.6 ± 15.2	128.8 ± 15.0	128.6 ± 15.0	127.8 ± 15.0	< 0.001
Fasting glucose (mg/dL)	143.8 ± 40.5	142.0 ± 38.0	142.4 ± 41.1	146.2 ± 50.7	< 0.001
Triglyceride (mg/dL)	141.9 (141.5–142.3)	146.7 (146.3–147.1)	149.7 (149.3–150.1)	148.8 (148.3–149.2)	< 0.001
HDL-C (mg/dL)	52.0 ± 21.8	51.8 ± 21.2	51.6 ± 21.1	51.7 ± 20.9	< 0.001
LDL-C (mg/dL)	111.6 ± 45.0	111.9 ± 44.4	111.4 ± 44.7	110.3 ± 46.5	< 0.001
GLU_SD (mg/dL)	12.4 ± 9.8	22.7 ± 14.1	32.3 ± 18.7	47.1 ± 25.3	< 0.001
GLU_CV (%)	8.1 ± 4.0	15.5 ± 5.0	22.9 ± 6.9	35.5 ± 11.9	< 0.001
GLU_VIM (%)	4.1 ± 1.8	9.6 ± 1.3	14.6 ± 1.7	24.3 ± 5.7	< 0.001
GLU_ARV (mg/dL)	15.2 ± 12.9	27.0 ± 18. 8	37.8 ± 25.2	52.4 ± 33.7	< 0.001
Current smoker (%)	33185 (21.3)	37301 (23.9)	42135 (27)	48736 (31.2)	< 0.001
Heavy drinking (%)	14997 (9.6)	15563 (10.0)	15755 (10.1)	14992 (9.6)	< 0.001
Regular exercise (%)	40718 (26.0)	38546 (24.7)	36142 (23.2)	32955 (21.1)	< 0.001
Comorbidities					
Hypertension (%)	89659 (57.5)	87808 (56.3)	84796 (54.3)	78148 (50.1)	< 0.001
Dyslipidemia (%)	60077 (38.5)	58915 (37.8)	56500 (36.2)	50461 (32.3)	< 0.001
CKD (%)	15740 (10.1)	15962 (10.2)	16598 (10.7)	17320 (11.1)	< 0.001
Heart disease (%)	6265 (4.6)	5786 (4.3)	5189 (4.0)	4564 (3.7)	< 0.001
Any malignancy (%)	4125 (2.6)	3930 (2.5)	3772 (2.4)	3892 (2.5)	0.001
Income (lower 20%, %)	34507 (22.1)	35412 (22.7)	37482 (24.0)	39595 (25.4)	< 0.001
Antidiabetic medication					
Metformin	84747 (54.3)	77947 (50.0)	72119 (46.2)	62185 (39.9)	< 0.001
Sulfonylurea	81607 (52.3)	77767 (49.8)	74710 (47.9)	66155 (42.4)	< 0.001
Meglitinide	4246 (2.7)	4050 (2.6)	3850 (2.5)	3785 (2.4)	< 0.001
Thiazolidinedione	14054 (9.0)	13354 (8.6)	12355 (7.9)	10933 (7.0)	<0.001
DPP-4 inhibitor	13802 (8.8)	12730 (8.2)	11766 (7.5)	9535 (6.1)	< 0.001
a-Glucosidase inhibitor	21803 (14.0)	20564 (13.2)	20055 (12.9)	18458 (11.8)	< 0.001
Insulin	10064 (6.5)	9936 (6.4)	10655 (6.8)	13320 (8.5)	< 0.001
Number of oral anti-diabetic medications					< 0.001
0	42298 (27.1)	51240 (32.8)	58967 (37.8)	72375 (46.4)	
1	38976 (25.0)	33797 (21.7)	29285 (18.8)	23217 (14.9)	
2	48766 (31.3)	45997 (29.5)	43216 (27.7)	38441 (24.6)	
≥ 3	26021 (16.7)	25024 (16.0)	24592 (15.8)	22025 (14.1)	
Duration of diabetes ≥5 years (%)	62489 (40.0)	55604 (35.6)	50193 (32.2)	43641 (28.0)	< 0.001
Type 1 diabetes (%)	3104 (2.0)	3214 (2.1)	3745 (2.4)	5548 (3.6)	< 0.001
Number of exams					< 0.001
3	126421 (81.0)	113451 (72.7)	108293 (69.4)	104468 (66.9)	
4	14692 (9.4)	19227 (12.3)	21858 (14.0)	24324 (15.6)	
5	14948 (9.6)	23380 (15.0)	25909 (16.6)	27266 (17.5)	
Time interval between adjacent exams (years)	1.8 ± 0.3	1.7 ± 0.3	1.7 ± 0.3	1.7 ± 0.3	<0.001

Q1:0–7.4; Q2:7.4–11.9; Q3:11.9–17.8; Q4:17.8–87.7. Data are presented as mean ± standard deviation, geometric mean (95% confidence interval), or number (%). One-way analysis of variance and Chi squared tests were used to compare the characteristics of the study subjects at baseline. Post-hoc multiple comparison analysis was performed with Bonferroni correction, and triglyceride levels were log-transformed for analysis. ARV, average real variability; BMI, body mass index; BP, blood pressure; CKD, chronic kidney disease; CV, coefficient of variation; HDL-C, high-density lipoprotein-cholesterol; LDL-C, low-density lipoprotein-cholesterol; SD, standard deviation; VIM, variability independent of mean; WC, waist circumference

During the median (interquartile range) follow-up period of 8.0 (7.3–8.4) years, 25,038 cases of stroke, 15,832 cases of MI, and 44,716 cases of death were identified. As shown in Table 2, the HRs for stroke, MI, and all-cause mortality serially increased as the FG VIM quartile increased. Subjects in FG VIM quartile 4 had a 20% higher risk for stroke and MI, and a 32% higher risk for all-cause mortality, versus those in quartile 1, even after adjustment for several risk factors and mean FG. We also tested the following GV calculation methods: SD, CV, and ARV. A similar relationship was obtained in FG SD, CV, and ARV quartiles instead of VIM (Additional file 1: Table S1).

**Table 2:** Hazard ratios (HRs) and 95% confidence intervals (CIs) for the incidence of stroke, myocardial infarction, and all-cause mortality by quartile of fasting glucose variability

	Events (n)	Follow-up duration (person-years)	Incidence rate (per 1000 person-years)	Age- and sex- Adjusted HR (95% CI)	Multivariate-adjusted HR (95% CI)	
					Model 1	Model 2
Stroke						
VIM Q1 (n = 156061)	6350	1185704.0	5.36	1 (Ref.)	1 (Ref.)	1 (Ref.)
VIM Q2 (n = 156058)	6162	1191326.6	5.17	1.03 (0.99–1.07)	1.03 (0.99–1.06)	1.06 (1.02–1.09)
VIM Q3 (n = 156060)	6214	1193013.0	5.21	1.08 (1.04–1.12)	1.05 (1.02–1.09)	1.10 (1.06–1.14)
VIM Q4 (n = 156058)	6312	1191670.8	5.30	1.17 (1.13–1.21)	1.10 (1.07–1.14)	1.20 (1.16–1.24)
*P* for trend				< 0.001	< 0.001	< 0.001
Myocardial infarction						
VIM Q1 (n = 156061)	3859	1194006.5	3.23	1 (Ref.)	1 (Ref.)	1 (Ref.)
VIM Q2 (n = 156058)	3899	1199121.6	3.25	1.06 (1.01–1.11)	1.05 (1.00–1.10)	1.07 (1.02–1.12)
VIM Q3 (n = 156060)	3978	1200620.1	3.31	1.11 (1.07–1.16)	1.09 (1.04–1.13)	1.12 (1.07–1.17)
VIM Q4 (n = 156058)	4096	1199009.4	3.42	1.21 (1.16–1.27)	1.14 (1.09–1.20)	1.20 (1.15–1.25)
*P* for trend				< 0.001	< 0.001	< 0.001
All-cause mortality						
VIM Q1 (n = 156061)	10506	1205853.5	8.71	1 (Ref.)	1 (Ref.)	1 (Ref.)
VIM Q2 (n = 156058)	10608	1211059.8	8.76	1.09 (1.06–1.119)	1.08 (1.05–1.11)	1.10 (1.07–1.13)
VIM Q3 (n = 156060)	11181	1212864.1	9.22	1.19 (1.16–1.22)	1.15 (1.12–1.18)	1.17 (1.14–1.20)
VIM Q4 (n = 156058)	12421	1211473.9	10.25	1.40 (1.36–1.44)	1.29 (1.26–1.33)	1.32 (1.29–1.36)
*P* for trend				< 0.001	< 0.001	< 0.001

Q1:0–7.4; Q2:7.4–11.9; Q3:11.9–17.8; Q4:17.8–87.7. Model 1 is adjusted for age, sex, body mass index, alcohol drinking, smoking, regular exercise, presence of hypertension, dyslipidemia, chronic kidney disease, and lower 20% income. Model 2 is the same as model 1, plus further adjustment for duration of diabetes over 5 years, the number of classes of oral anti-diabetic medication taken in the 12 months prior to baseline, presence of prescription history of insulin, and mean of fasting glucose. VIM, variability independent of mean

**Fig. 3:**
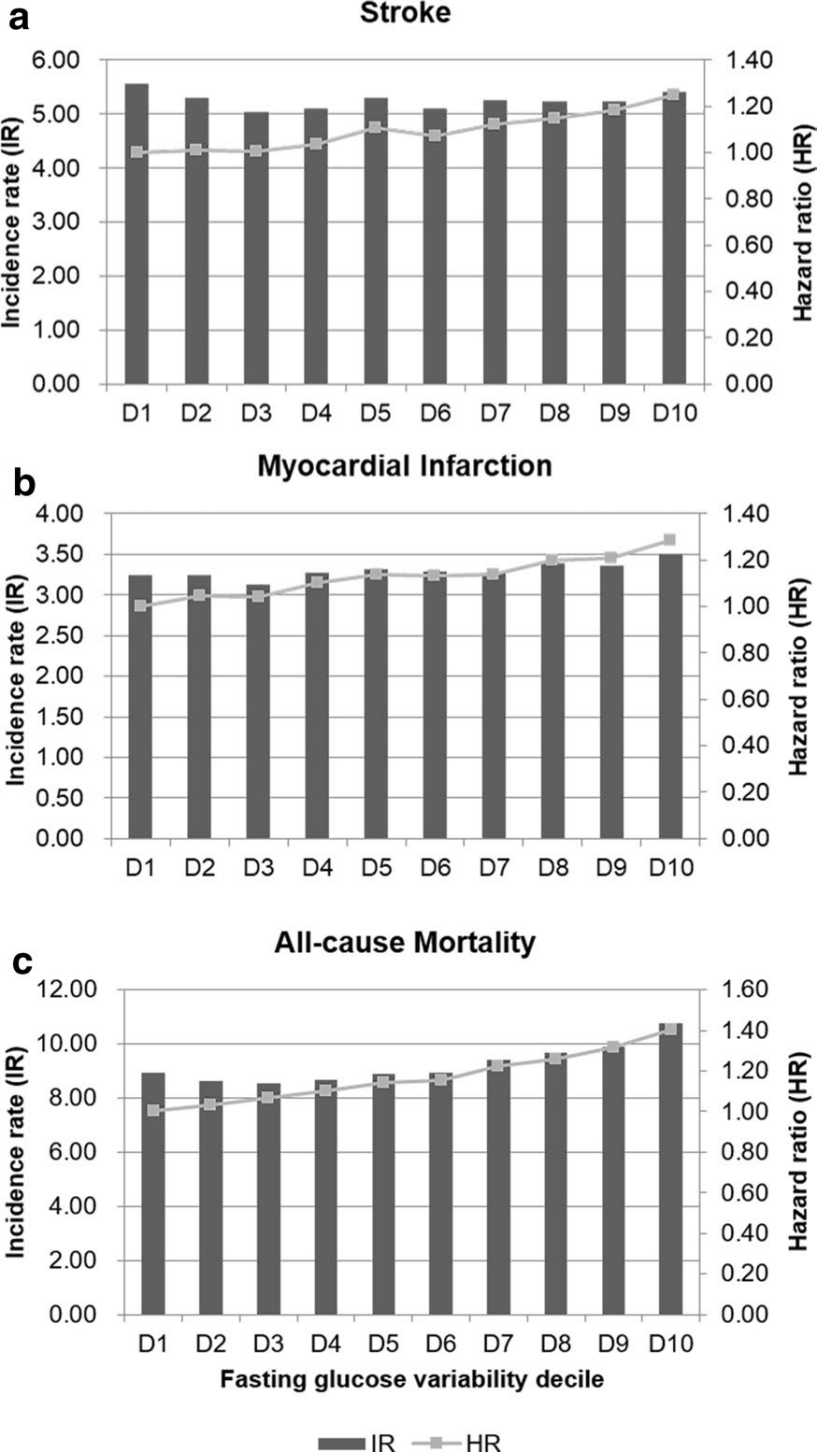
Hazard ratios (HRs) and incidence rates of (**a**) stroke, (**b**) myocardial infarction, and (**c**) all-cause mortality by deciles of fasting glucose variability, assessed by variability independent of mean

When we divided individuals into FG VIM deciles, the risks of stroke, MI, and all-cause death showed a positive dose–response association with the FG VIM decile (Fig. 3 and Additional file 1: Table S2).

In the subgroup analyses, FG VIM quartile 4 showed a consistently increased risk for all-cause mortality (Tables 3 and 4) except in young individuals aged 20-39. A similar finding was observed for stroke and MI, with exceptions in younger individuals and those with malignancy, shorter diabetes duration, baseline FG ≥ 126 mg/dL, T1DM, and treatment with thiazolidinedione and insulin.

**Table 3:** Subgroup analysis according to clinically relevant factors in quartile 4 versus fasting glucose variability of quartiles 1–3

	Stroke		Myocardial infarction		All-cause mortality	
	IR per 1000	HR (95% CI)	IR per 1000	HR (95% CI)	IR per 1000	HR (95% CI)
Age (years)						
20–39 (n = 51687)	0.56	0.78 (0.59–1.03)	0.93	0.89 (0.72–1.09)	1.14	1.01 (0.84–1.22)
40–64 (n = 413136)	3.43	1.04 (0.99–1.09)	2.50	1.07 (1.01–1.12)	4.82	1.17 (1.13–1.21)
≥ 65 (n = 159414)	12.08	1.13 (1.09–1.18)	6.35	1.12 (1.07–1.18)	24.15	1.18 (1.15–1.21)
*P* for interaction		0.001		0.056		0.102
Sex						
Male (n = 411054)	5.07	1.05 (1.01–1.09)	3.29	1.06 (1.01–1.11)	10.15	1.17 (1.14–1.20)
Female (n = 213183)	5.62	1.13 (1.07–1.18)	3.33	1.13 (1.07–1.21)	7.49	1.19 (1.14–1.24)
*P* for interaction		0.028		0.071		0.387
BMI						
< 25 kg/m^2^ (n = 331714)	5.66	1.10 (1.06–1.14)	3.44	1.08 (1.03–1.13)	11.31	1.19 (1.16–1.22)
≥ 25 kg/m^2^ (n = 292523)	4.81	1.05(1.01–1.10)	3.15	1.10 (1.04–1.16)	6.91	1.14 (1.10–1.18)
*P* for interaction		0.117		0.477		0.020
Current smoking						
No (n = 462880)	5.33	1.13 (1.09–1.17)	3.23	1.12 (1.07–1.17)	9.11	1.19 (1.16–1.22)
Yes (n = 161357)	5.06	0.94 (0.89–0.99)	3.53	1.01 (0.95–1.08)	9.59	1.15 (1.10–1.19)
*P* for interaction		<0.001		0.026		0.089
Hypertension						
No (n = 283826)	3.35	1.02 (0.97–1.08)	2.38	1.01 (0.95–1.08)	6.50	1.17 (1.13–1.21)
Yes (n = 340411)	6.89	1.11 (1.07–1.14)	4.09	1.13 (1.09–1.19)	11.55	1.19 (1.16–1.22)
*P* for interaction		0.011		0.001		0.845
Dyslipidemia						
No (n = 398284)	5.24	1.05 (1.01–1.09)	3.09	1.09 (1.04–1.14)	9.99	1.17 (1.15–1.20)
Yes (n = 225953)	5.29	1.13 (1.07–1.18)	3.68	1.10 (1.04–1.16)	7.93	1.19 (1.15–1.24)
*P* for interaction		0.007		0.397		0.476
Malignancy						
No (n = 608518)	5.25	1.15 (1.11–1.18)	3.39	1.13 (1.09–1.18)	9.75	1.24 (1.21–1.27)
Yes (n = 15719)	7.22	1.03 (0.88–1.22)	4.68	1.11 (0.90–1.36)	32.14	1.18 (1.09–1.28)
*P* for interaction		0.213		0.682		0.702
Income lower 20%						
No (n = 477241)	5.08	1.08 (1.05–1.12)	3.22	1.10 (1.05–1.14)	8.82	1.21 (1.18–1.24)
Yes (n = 146996)	5.83	1.07 (1.01–1.13)	3.57	1.07 (1.00–1.15)	10.57	1.12 (1.08–1.17)
*P* for interaction		0.759		0.618		0.001

Adjusted for age, sex, body mass index, alcohol drinking, smoking, regular exercise, presence of hypertension, dyslipidemia, chronic kidney disease, and lower 20% income, duration of diabetes over 5 years, the number of classes of oral anti-diabetic medication taken in the 12 months prior to baseline, presence of prescription history of insulin, and mean of fasting glucose

**Table 4:** Subgroup analysis according to the characteristics of diabetes in quartile 4 versus fasting glucose variability of quartiles 1–3

	Stroke		Myocardial infarction		All-cause mortality	
	IR per 1000	HR (95% CI)	IR per 1000	HR (95% CI)	IR per 1000	HR (95% CI)
Duration of diabetes^a^						
< 5 years (n = 412310)	4.02	1.01 (0.97–1.06)	2.63	1.03 (0.98–1.08)	7.41	1.16 (1.12–1.19)
≥ 5 years (n = 211927)	7.74	1.17 (1.12–1.22)	4.66	1.17 (1.11–1.24)	12.87	1.22 (1.18–1.26)
*P* for interaction		< 0.001		0.001		0.011
Baseline fasting glucose^a^						
< 126 mg/dL (n = 188779)	6.30	1.21 (1.15–1.27)	3.94	1.16 (1.09–1.23)	11.64	1.17 (1.13–1.21)
≥ 126 mg/dL (n = 435458)	4.81	0.95 (0.92–0.99)	3.03	1.01 (0.96–1.06)	8.20	1.11 (1.08–1.14)
*P* for interaction		< 0.001		< 0.001		< 0.001
Subtype of diabetes^a^						
T2DM (n = 608626)	5.02	1.14 (1.11–1.17)	3.26	1.14 (1.10–1.19)	9.70	1.22(1.19–1.25)
T1DM (n = 15611)	13.49	1.11 (0.99–1.24)	8.01	0.96 (0.84–1.11)	26.23	1.25(1.15–1.35)
*P* for interaction		0.267		0.065		0.186
Metformin^b^						
No (n = 327239)	4.05	1.17 (1.12–1.22)	2.48	1.18 (1.12–1.25)	8.59	1.30 (1.26–1.34)
Yes (n = 296998)	7.22	1.18 (1.13–1.22)	4.72	1.16 (1.11–1.22)	12.80	1.25 (1.22–1.29)
*P* for interaction		0.622		0.976		0.150
Sulfonylurea^b^						
No (n = 323998)	3.56	1.19 (1.13–1.24)	2.48	1.18 (1.12–1.25)	7.42	1.30 (1.26–1.34)
Yes (n = 300239)	7.72	1.17 (1.13–1.21)	4.72	1.16 (1.11–1.22)	14.16	1.26 (1.23–1.30)
*P* for interaction		0.858		0.976		0.380
Meglitinide^b^						
No (n = 608306)	5.19	1.17 (1.13–1.20)	3.34	1.16 (1.12–1.20)	10.02	1.27 (1.25–1.30)
Yes (n = 15931)	10.02	1.28 (1.11–1.48)	6.51	1.37(1.15–1.64)	20.14	1.37 (1.24–1.52)
*P* for interaction		0.139		0.056		0.080
Thiazolidinedione^b^						
No (n = 573541)	5.21	1.17 (1.14–1.21)	3.37	1.17 (1.13–1.22)	10.17	1.28 (1.25–1.31)
Yes (n = 50696)	6.44	1.17 (1.06–1.29)	3.97	1.13 (0.99–1.28)	11.37	1.25 (1.16–1.34)
*P* for interaction		0.969		0.561		0.526
DPP-4 inhibitor^b^						
No (n = 576404)	5.25	1.17 (1.13–1.20)	3.37	1.17 (1.13–1.21)	10.23	1.27 (1.25–1.30)
Yes (n = 47833)	6.06	1.21 (1.09–1.35)	4.06	1.18 (1.03–1.35)	10.63	1.35 (1.24–1.46)
*P* for interaction		0.287		0.835		0.079
a-Glucosidase inhibitor^b^						
No (n = 543357)	4.70	1.15 (1.12–1.19)	3.09	1.15 (1.10–1.20)	9.29	1.28 (1.25–1.31)
Yes (n = 80880)	9.92	1.23 (1.16–1.32)	5.94	1.24 (1.14–1.34)	17.62	1.25 (1.19–1.31)
*P* for interaction		0.053		0.157		0.519
Insulin^b^						
No (n = 580262)	4.75	1.15 (1.11–1.18)	3.05	1.14 (1.10–1.19)	9.02	1.24 (1.22–1.27)
Yes (n = 43975)	11.68	1.11 (1.03–1.19)	7.68	1.08 (0.99–1.18)	24.31	1.19 (1.13–1.25)
*P* for interaction		0.874		0.568		0.492

^a^Adjusted for age, sex, body mass index, alcohol drinking, smoking, regular exercise, presence of hypertension, dyslipidemia, chronic kidney disease, and lower 20% income, duration of diabetes over 5 years, the number of classes of oral anti-diabetic medication taken in the 12 months prior to baseline, presence of prescription history of insulin, and mean of fasting glucose

^b^Adjusted for age, sex, body mass index, alcohol drinking, smoking, regular exercise, presence of hypertension, dyslipidemia, chronic kidney disease, and lower 20% income, duration of diabetes over 5 years, and mean of fasting glucose

**Fig. 4:**
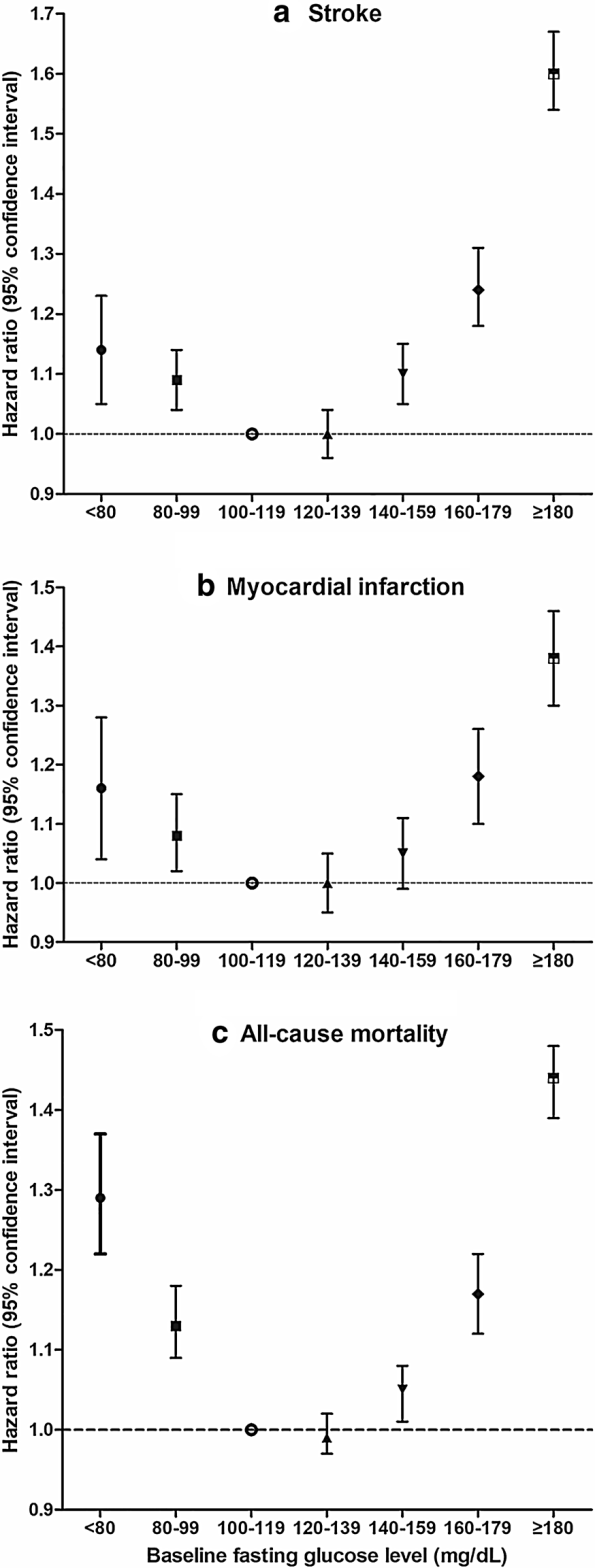
Hazard ratios (HRs) of (**a**) stroke, (**b**) myocardial infarction, and (**c**) all-cause mortality according to baseline fasting glucose level

On the other hand, the relationship between FG status and the risk of clinical outcomes showed a U-shaped association (Fig. 4 and Additional file 1: Table S3). Compared to individuals whose FG levels were in the range 100-119 mg/dL, those with FG < 100 mg/dL or ≥ 160 mg/dL had significantly higher HRs. This relationship was consistent after dividing the subjects by prescription of anti-diabetic medications (Additional file 1: Table S4).

## Discussion

This nationwide population-based study in diabetes revealed that long-term GV for 5 years predicted future development of stroke, MI, and all-cause death, independent of anti-diabetic medication, metabolic risk factors, and mean fasting glucose. This association showed a linear fashion, compared to the U-shaped association between FG and outcomes. In the subgroup analysis, the impact of GV was higher in the elderly and those with a longer duration of diabetes and lower FG levels.

### Long-term glucose variability and clinical outcomes

Several medium-sized cohort studies have reported the impact of long-term GV [6, 15, 17–21]. Variability in FG is an independent predictor of all-cause mortality, and the highest tertile group in 1400 type 2 diabetes patients included in the VERONA study had a 67% higher risk [17, 21]. In the sub-analysis of the Action in Diabetes and Vascular Disease: Preterax and Diamicron MR Controlled Evaluation (ADVANCE) trial, variability in FG was associated with an increased risk of death from cardiovascular causes, nonfatal MI, or nonfatal stroke [15]. Furthermore, GV might reflect better the impact of glycemic control on diabetic complications than HbA1c, or at least, GV can complement the power of HbA1c in risk prediction [7, 14, 15]. In the ADVANCE trial, HRs (95% CIs) estimated for each 10-percentile point increase in SD of FG and HbA1c were 1.12 (1.08–1.16) and 1.05 (1.01–1.09), respectively [15].

However, these studies have limitations such as short follow-up period [15] and a relatively small population size, and the impact of GV was only shown in some subgroups, but not the entire population [18]. In the present study, we computed long-term GV over 5 years and assessed risk for a median of 8 years of follow-up. In addition, this dataset includes almost all Korean diabetic patients. In this respect, the worst outcome in the highest GV quartile even after adjustment for mean FG provides a clear indication that GV can be considered as a new risk factor for stroke, MI, and all-cause mortality.

### Interpretation of the impact of glucose variability

A few mechanisms might be involved in the relationship between FG variability and clinical outcomes. Transient high glucose spikes have been shown to impair endothelial function [28, 29], increase oxidative stress more than sustained chronic hyperglycemia [30, 31], and induce β-cell dysfunction [12]. Furthermore, higher GV was strongly associated with increased lipid level and decreased fibrous content with a larger plaque burden [32].

In subgroup analysis of this study, the impact of GV was greater in the elderly and those with a longer duration of diabetes and FG level < 126 mg/dL. These findings were partially in accordance with previous publications [18, 20]. In the Verona Diabetes Study, the positive association between GV and mortality risk was confined to patients older than 65 years [18]. The elderly are known to be more susceptible to oxidative stress than younger individuals because their defense mechanisms are less efficient [19]. The limited significance seen in current smokers could be explained by the higher proportion of young subjects in the current smoker group. In the same notion, patients with a longer diabetes duration and lower FG might reflect a population that is more vulnerable to GV. It is interesting that contrary to other studies [15, 19, 20], GV had a greater impact among lower FG patients than in higher FG patients, which may be due to an ethnic difference, but the reason is difficult to ascertain from this study. As we also showed that lower FG level was significantly associated with more CVD and death events, the higher GV in the lower FG group augmented the risk for future CVD and death, indicating the target population for reducing these outcomes.

In addition to this pathophysiologic interpretation, elevated GV might be an indicator of irregular compliance to therapy, comorbidity, poor health, or diabetic complications resulting in the increase of mortality [17]. However, contrary to other studies [14, 17, 20], our study population with higher GV had a shorter duration of diabetes, had fewer comorbidities, and about half of them were not treated with anti-diabetic medication. Although there was a greater proportion of insulin users, the contribution of insulin users to the outcome was small since less than 10% of the study population was treated with insulin. We speculate that, compared to other hospital-based studies, this study included more low-risk diabetic patients since diagnosis of diabetes was based on not only health insurance claims data, but also general health screening exams, which resulted in detection of undiagnosed mild diabetic cases. Therefore, we can evaluate the impact of GV itself, rather than combined poor health status and comorbidities as in other studies.

In subgroup analysis according to anti-diabetic medications, interaction analysis did not find any significance, contrary to previous studies which showed sulfonylurea increases glucose fluctuation and risk of hypoglycemia [33], and DPP-4 inhibitors and the novel insulin analogue degludec reduced GV [34–36]. Given that actual drug exposure could not be determined because of the retrospective study design, a future well-designed, randomized controlled trial is expected to resolve this concern.

### Assessment of glycemic variability

There are no standard indices for quantification of long-term glycemic variability [37], so each value has distinct characteristics. The SD reflects dispersion of measurements around the mean and is sensitive to low sampling frequency, while CV is a standardized variation providing direct comparison among study groups. The ARV index, which averages the absolute differences between successive measurements, might be a reliable index for time series variability [38, 39]. However, SD, CV, and ASV are partially dependent on the mean and its changes over time, and this may not be resolved even if adjusted for mean value [40]. On the other hand, VIM is a measure of variability designed not to correlate with mean level [24, 41] but is sample-specific [42, 43]. Therefore, we used VIM as the main measurement of FG variability, and similar results to other estimates of glycemic variation support the robustness of our study (Additional file 1: Table S1).

### Limitations of this study

This is the first large-scale epidemiologic study demonstrating the impact of long-term variability in FG on CVD outcomes separately and all-cause death with a long-term follow-up of 8.0 (7.3–8.4) years. However, we are aware of several limitations of this study. First, given that HbA1c or postprandial glucose level was not measured in this study, incident diabetes might be underestimated. To enhance the accuracy of diabetes diagnosis, we combined ICD-10 codes and patient prescription histories in addition to fasting glucose level. Second, possible selection bias due to extracting patients based on the number of health check-ups is one of the limitations. To overcome it, we conducted various subgroup analyses. Finally, classification of T1DM based on the presence of ICD-10 E10 with at least one prescription of insulin might not be accurate. Because ICD-10 code is based on claims data, but autoantibody, insulin, or c-peptide level was not measured, the prevalence of T1DM could be overestimated. However, we performed subgroup analysis after stratifying the subjects according to subtype of diabetes. There was a significance in all-cause mortality in T1DM. The absence of significance in stroke and MI was thought to be related to the small number of population.

## Conclusion

This nationwide population-based study with long-term follow-up showed that GV had a dose–response relationship with the risk of stroke, MI, and all-cause mortality in diabetes, especially in the elderly and those with a longer duration of diabetes and lower FG levels. This important health issue will play a role in reducing future development of CVD and death attributed to the increasing population of diabetes.

## Supplementary information

**Additional file 1: Table S1.** Hazard ratios (HRs) and 95% confidence intervals (CIs) for the incidence of stroke, myocardial infarction, and all-cause mortality by quartiles of fasting glucose variability, assessed by standard deviation, coefficient of variation, and average real variability. **Table S2.** Hazard ratios (HRs) and 95% confidence intervals (CIs) for the incidence of stroke, myocardial infarction, and all-cause mortality by deciles of fasting glucose variability. **Table S3.** Hazard ratios (HRs) and 95% confidence intervals (CIs) for the incidence of stroke, myocardial infarction, and all-cause mortality according to baseline fasting glucose level. **Table S4.** Hazard ratios (HRs) and 95% confidence intervals (CIs) according to baseline fasting glucose level and antidiabetic medication (ADM).

## Data Availability

The data that support the findings of this study are available from the National Health Insurance Corporation but restrictions apply to the availability of these data, which were used under license for the current study, and so are not publicly available. Data are however available from the authors upon reasonable request and with permission of the National Health Insurance Corporation.

## Abbreviations

ARV: Average real variability
BMI: Body mass index
BP: Blood pressure
CI: Confidence interval
CKD: Chronic kidney disease
CV: Coefficient of variation
CVD: Cardiovascular disease
DPP-4 inhibitors: Inhibitors of dipeptidyl peptidase 4
eGFR: Estimated glomerular filtration rate
FG: Fasting glucose
GV: Glucose variability
HR: Hazard ratio
ICD-10: International Classification of Diseases, tenth revision
LDL-C: low-density lipoprotein cholesterol
MI: Myocardial infarction
NHIC: National Health Insurance System
SD: Standard deviation
T1DM: Type 1 diabetes
T2DM: Type 2 diabetes
VIM: Variability independent of the mean
