# Supplementary material for: Glucose variability and the risks of stroke, myocardial infarction, and all-cause mortality in individuals with diabetes: retrospective cohort study
Source: Cardiovasc Diabetol. 2020 Sep 22;19:144. doi: 10.1186/s12933-020-01134-0 (PMC7510288; doi:10.1186/s12933-020-01134-0)
Supplement: Supplementary file 1 — Additional file 1: Table S1. Hazard ratios (HRs) and 95% confidence intervals (CIs) for the incidence of stroke, myocardial infarction, and all-cause mortality by quartiles of fasting glucose variability, assessed by standard deviation, coefficient of variation, and average real variability. Table S2. Hazard ratios (HRs) and 95% confidence intervals (CIs) for the incidence of stroke, myocardial infarction, and all-cause mortality by deciles of fasting glucose variability. Table S3. Hazard ratios (HRs) and 95% confidence intervals (CIs) for the incidence of stroke, myocardial infarction, and all-cause mortality according to baseline fasting glucose level. Table S4. Hazard ratios (HRs) and 95% confidence intervals (CIs) according to baseline fasting glucose level and antidiabetic medication (ADM). [file 12933_2020_1134_MOESM1_ESM.docx]

**Table S1. Hazard ratios (HRs) and 95% confidence intervals (CIs) for the incidence of stroke, myocardial infarction, and all-cause mortality by quartiles of fasting glucose variability, assessed by standard deviation, coefficient of variation, and average real variability**

|  | **Stroke** | | |  | **Myocardial infarction** | | |  | **Death** | | |
| --- | --- | --- | --- | --- | --- | --- | --- | --- | --- | --- | --- |
|  | **Events**  **(n)** | **Incidence rate**  **(per 1000**  **person-years)** | **Multivariate-adjusted HR**  **(95% CI)** |  | **Events**  **(n)** | **Incidence rate**  **(per 1000**  **person-years)** | **Multivariate-adjusted HR**  **(95% CI)** |  | **Events**  **(n)** | **Incidence rate**  **(per 1000**  **person-years)** | **Multivariate-adjusted HR**  **(95% CI)** |
| **FG SD Quartiles** | |  |  |  |  |  |  |  |  |  |  |
| Q1 (n=156060) | 5854 | 4.93 | 1 (Ref.) |  | 3650 | 3.05 | 1 (Ref.) |  | 10746 | 8.91 | 1 (Ref.) |
| Q2 (n=155929) | 5630 | 4.71 | 1.08 (1.04–1.12) |  | 3623 | 3.01 | 1.09 (1.04–1.14) |  | 10005 | 8.24 | 1.06 (1.03–1.09) |
| Q3 (n=156222) | 5811 | 4.85 | 1.11 (1.07–1.15) |  | 3797 | 3.15 | 1.14 (1.09–1.19) |  | 10507 | 8.64 | 1.13 (1.10–1.17) |
| Q4 (n= 156026) | 7743 | 6.56 | 1.22 (1.17–1.27) |  | 4762 | 4.00 | 1.21 (1.15–1.27) |  | 13458 | 11.17 | 1.31 (1.27–1.35) |
| *P* for trend |  |  | <0.001 |  |  |  | <0.001 |  |  |  | <0.001 |
| **FG CV Quartiles** | |  |  |  |  |  |  |  |  |  |  |
| Q1 (n=156056) | 6016 | 5.07 | 1 (Ref.) |  | 3649 | 3.05 | 1 (Ref.) |  | 10535 | 8.74 | 1 (Ref.) |
| Q2 (n=156058) | 5835 | 4.88 | 1.05 (1.01–1.09) |  | 3864 | 3.22 | 1.13 (1.08–1.18) |  | 10330 | 8.51 | 1.08 (1.05–1.11) |
| Q3 (n=156064) | 6032 | 5.05 | 1.12 (1.08–1.16) |  | 3806 | 3.16 | 1.13 (1.08–1.18) |  | 10772 | 8.87 | 1.16 (1.13–1.20) |
| Q4 (n=156059) | 7155 | 6.04 | 1.19 (1.15–1.23) |  | 4513 | 3.78 | 1.21 (1.16–1.27) |  | 13079 | 10.83 | 1.30 (1.26–1.33) |
| *P* for trend |  |  | <0.001 |  |  |  | <0.001 |  |  |  | <0.001 |
| **FG ARV Quartiles** | |  |  |  |  |  |  |  |  |  |  |
| Q1 (n=155869) | 5589 | 4.69 | 1(Ref.) |  | 3519 | 2.93 | 1(Ref.) |  | 10118 | 8.36 | 1(Ref.) |
| Q2 (n=154314) | 5343 | 4.51 | 1.05 (1.01–1.09) |  | 3474 | 2.92 | 1.07 (1.02–1.12) |  | 9644 | 8.02 | 1.05 (1.02–1.08) |
| Q3 (n=157455) | 6072 | 5.05 | 1.10 (1.06–1.14) |  | 3964 | 3.27 | 1.13 (1.08–1.19) |  | 11088 | 9.07 | 1.15 (1.12–1.18) |
| Q4 (n=156599) | 8034 | 6.81 | 1.20 (1.16–1.25) |  | 4875 | 4.09 | 1.19 (1.13–1.25) |  | 13866 | 11.50 | 1.29 (1.25–1.32) |
| *P* for trend |  |  | <0.001 |  |  |  | <0.001 |  |  |  | <0.001 |

Adjusted for age, sex, body mass index, alcohol drinking, smoking, regular exercise, presence of hypertension, dyslipidemia, chronic kidney disease, lower 20% income, duration of diabetes over 5 years, the number of classes of oral anti-diabetic medication taken in the 12 months prior to baseline, presence of prescription history of insulin, and mean of fasting glucose. FG, fasting glucose; SD, standard deviation; CV, coefficient of variation; ARV, average real variability.

**Table S2. Hazard ratios (HRs) and 95% confidence intervals (CIs) for the incidence of stroke, myocardial infarction, and all-cause mortality by deciles of fasting glucose variability^a^**

| **Group** | **Events (n)** | **Follow-up**  **duration**  **(person-years)** | **Incidence rate**  **(per 1000**  **person-years)** | **Multivariate-adjusted**  **HR (95% CI)** |
| --- | --- | --- | --- | --- |
|  |  |  |  |  |
| **Stroke** | |  |  |  |
| VIM D1 (n=62421) | 2625 | 472129.6 | 5.56 | 1(Ref.) |
| VIM D2 (n=62425) | 2523 | 475124.4 | 5.31 | 1.01 (0.96–1.07) |
| VIM D3 (n=62425) | 2402 | 476135.6 | 5.04 | 1.01 (0.95–1.06) |
| VIM D4 (n=62424) | 2433 | 476716.4 | 5.10 | 1.04 (0.98–1.10) |
| VIM D5 (n=62424) | 2529 | 476924.6 | 5.30 | 1.11 (1.05–1.17) |
| VIM D6 (n=62424) | 2442 | 477421.7 | 5.11 | 1.07 (1.01–1.13) |
| VIM D7 (n=62423) | 2510 | 476890.1 | 5.26 | 1.12 (1.06–1.19) |
| VIM D8 (n=62424) | 2500 | 476909.6 | 5.24 | 1.15 (1.09–1.22) |
| VIM D9 (n=62424) | 2499 | 477065.9 | 5.24 | 1.18 (1.12–1.25) |
| VIM D10 (n=62423) | 2575 | 476396.7 | 5.41 | 1.25 (1.18–1.32) |
| *P* for trend |  |  |  | <0.001 |
| **Myocardial infarction** | |  |  |  |
| VIM D1 (n=62421) | 1540 | 475799.5 | 3.24 | 1(Ref.) |
| VIM D2 (n=62425) | 1555 | 478398.8 | 3.25 | 1.05 (0.98–1.12) |
| VIM D3 (n=62425) | 1497 | 479202 | 3.12 | 1.04 (0.97–1.12) |
| VIM D4 (n=62424) | 1572 | 479712.8 | 3.28 | 1.10 (1.03–1.18) |
| VIM D5 (n=62424) | 1594 | 480015.1 | 3.32 | 1.14 (1.06–1.22) |
| VIM D6 (n=62424) | 1581 | 480417 | 3.29 | 1.13 (1.06–1.22) |
| VIM D7 (n=62423) | 1573 | 480037 | 3.28 | 1.14 (1.06–1.22) |
| VIM D8 (n=62424) | 1627 | 479878.8 | 3.39 | 1.20 (1.12–1.29) |
| VIM D9 (n=62424) | 1611 | 480183.8 | 3.35 | 1.21 (1.13–1.30) |
| VIM D10 (n=62423) | 1682 | 479112.8 | 3.51 | 1.29 (1.20–1.38) |
| *P* for trend | |  |  | <0.001 |
| **All-cause mortality** | |  |  |  |
| VIM D1 (n=62421) | 4285 | 480567.4 | 8.92 | 1(Ref.) |
| VIM D2 (n=62425) | 4177 | 483050.0 | 8.65 | 1.03 (0.99–1.08) |
| VIM D3 (n=62425) | 4137 | 483821.8 | 8.55 | 1.06 (1.02–1.11) |
| VIM D4 (n=62424) | 4211 | 484490.6 | 8.69 | 1.10 (1.05–1.15) |
| VIM D5 (n=62424) | 4304 | 484983.5 | 8.87 | 1.14 (1.10–1.19) |
| VIM D6 (n=62424) | 4339 | 485251.2 | 8.94 | 1.15 (1.10–1.20) |
| VIM D7 (n=62423) | 4574 | 484854.4 | 9.43 | 1.22 (1.17–1.27) |
| VIM D8 (n=62424) | 4686 | 484978.1 | 9.66 | 1.26 (1.21–1.31) |
| VIM D9 (n=62424) | 4796 | 485005.7 | 9.89 | 1.32 (1.26–1.37) |
| VIM D10 (n=62423) | 5207 | 484248.5 | 10.75 | 1.40 (1.35–1.46) |
| *P* for trend |  |  |  | <0.001 |

^a^D1:0–4.5; D2:4.5–6.5; D3:6.5–8.3; D4:8.3–10.0; D5:10.0–11.9; D6:11.9–13.9; D7:13.9–16.3; D8:16.3–19.5; D9:19.5–24.2; D10:24.2–87.7.

Adjusted for age, sex, body mass index, alcohol drinking, smoking, regular exercise, presence of hypertension, dyslipidemia, chronic kidney disease, and lower 20% income, duration of diabetes over 5 years, the number of classes of oral anti-diabetic medication taken in the 12 months prior to baseline, presence of prescription history of insulin, and mean fasting glucose. VIM, variability independent of mean.

| **Table S3. Hazard ratios (HRs) and 95% confidence intervals (CIs) for the incidence of** **stroke, myocardial infarction, and all-cause mortality** **according to baseline fasting glucose level** | | | | | | |
| --- | --- | --- | --- | --- | --- | --- |
|  | **Events (n)** | **Follow-up**  **duration**  **(person-years)** | **Incidence rate**  **(per 1000**  **person-years)** | **Age- and sex-**  **adjusted**  **HR (95% CI)** | **Multivariate-adjusted**  **HR (95% CI)** | |
|  |  |  |  |  | **Model 1** | **Model 2** |
| **Stroke** | |  |  |  |  |  |
| <80 mg/dL (n=10607) | 644 | 78343.4 | 8.22 | 1.32 (1.22–1.44) | 1.25 (1.15–1.36) | 1.14 (1.05–1.23) |
| 80–99 mg/dL (n=50502) | 2642 | 378495.2 | 6.98 | 1.12 (1.07–1.18) | 1.11 (1.05–1.16) | 1.09 (1.04–1.14) |
| 100–119 mg/dL (n=92770) | 4164 | 701909.3 | 5.93 | 1(Ref.) | 1(Ref.) | 1(Ref.) |
| 120–139 mg/dL (n=208,797) | 6,709 | 1,606,990.5 | 4.17 | 0.92 (0.88–0.96) | 0.93 (0.89–0.96) | 1.00 (0.96–1.04) |
| 140–159 mg/dL (n=116,653) | 4,153 | 895,758.4 | 4.64 | 1.05 (1.01–1.10) | 1.05 (1.01–1.10) | 1.10 (1.05–1.15) |
| 160–179 mg/dL(n=54752) | 2265 | 417768.2 | 5.42 | 1.25 (1.19–1.32) | 1.25 (1.19–1.32) | 1.24 (1.18–1.31) |
| ≥180 mg/dL (n=90156) | 4461 | 682449.4 | 6.54 | 1.74 (1.66–1.81) | 1.70 (1.63–1.78) | 1.60 (1.54–1.67) |
| **Myocardial infarction** | |  |  |  |  |  |
| <80 mg/dL (n=10607) | 419 | 79031.1 | 5.30 | 1.39 (1.25–1.54) | 1.29 (1.17–1.44) | 1.16 (1.04–1.28) |
| 80–99 mg/dL (n=50502) | 1635 | 381831.3 | 4.28 | 1.13 (1.06–1.20) | 1.10 (1.04–1.17) | 1.08 (1.02–1.15) |
| 100–119 mg/dL (n=92770) | 2615 | 707673.2 | 3.70 | 1(Ref.) | 1(Ref.) | 1(Ref.) |
| 120–139 mg/dL (n=208,797) | 4,397 | 1,614,309.4 | 2.72 | 0.90 (0.86–0.95) | 0.93 (0.89–0.98) | 1.00 (0.95–1.05) |
| 140–159 mg/dL (n=116,653) | 2,634 | 900,631.2 | 2.92 | 0.98 (0.93–1.04) | 1.01 (0.96–1.07) | 1.05 (0.99–1.11) |
| 160–179 mg/dL(n=54752) | 1432 | 420508.2 | 3.41 | 1.16 (1.09–1.24) | 1.19 (1.11–1.27) | 1.18 (1.10–1.26) |
| ≥180 mg/dL (n=90156) | 2700 | 688773.2 | 3.92 | 1.47 (1.40–1.56) | 1.47 (1.39–1.55) | 1.38 (1.30–1.46) |
| **All-cause mortality** | |  |  |  |  |  |
| <80 mg/dL (n=10607) | 1449 | 80287.5 | 18.05 | 1.58 (1.49–1.67) | 1.44 (1.36–1.52) | 1.29 (1.22–1.37) |
| 80–99 mg/dL (n=50502) | 5152 | 386822.2 | 13.32 | 1.20 (1.16–1.25) | 1.16 (1.12–1.20) | 1.13 (1.09-1.18) |
| 100–119 mg/dL (n=92770) | 7528 | 715565.3 | 10.52 | 1(Ref.) | 1(Ref.) | 1(Ref.) |
| 120–139 mg/dL (n=208,797) | 12,379 | 1,628,023.1 | 7.60 | 0.96 (0.93–0.99) | 0.95 (0.92–0.98) | 0.99 (0.97–1.02) |
| 140–159 mg/dL (n=116,653) | 7,242 | 908,742.5 | 7.97 | 1.04 (1.00–1.07) | 1.02 (0.98–1.05) | 1.05 (1.01–1.08) |
| 160–179 mg/dL(n=54752) | 3822 | 425023.0 | 8.99 | 1.20 (1.16–1.25) | 1.17 (1.12–1.22) | 1.17 (1.12–1.22) |
| ≥180 mg/dL (n=90156) | 7144 | 696787.7 | 10.25 | 1.62 (1.57–1.68) | 1.52 (1.47–1.57) | 1.44 (1.39–1.48) |

Model 1 is adjusted for age, sex, body mass index, alcohol drinking, smoking, regular exercise, presence of hypertension, dyslipidemia, chronic kidney disease, and lower 20% income.

Model 2 is the same as model 1, plus further adjustment for duration of diabetes over 5 years, the number of classes of oral anti-diabetic medication taken in the 12 months prior to baseline, and prescription history of insulin.

**Table S4. Hazard ratios (HRs) and 95% confidence intervals (CIs) according to baseline fasting glucose level and antidiabetic medication (ADM)**

|  | **Number of subjects** | | **Multivariate-adjusted HR (95% CI)^1^** | | | | | |
| --- | --- | --- | --- | --- | --- | --- | --- | --- |
|  |  |  | **Stroke** | | **Myocardial infarction** | | **All-cause mortality** | |
|  | **No ADM** | **ADM** | **No ADM** | **ADM** | **No ADM** | **ADM** | **No ADM** | **ADM** |
| Metformin |  |  |  |  |  |  |  |  |
| <80 mg/dL | 3597 | 7010 | 1.17(1.01–1.36) | 1.26(1.14–1.39) | 1.10(0.91–1.33) | 1.35(1.19–1.53) | 1.55(1.41–1.70) | 1.38(1.28–1.48) |
| 80–99 mg/dL | 15977 | 34525 | 1.13(1.04–1.23) | 1.10(1.03–1.16) | 1.13(1.02–1.26) | 1.09(1.01–1.17) | 1.21(1.14–1.28) | 1.14(1.09–1.19) |
| 100–119 mg/dL | 26222 | 66548 | 1(Ref.) | 1(Ref.) | 1(Ref.) | 1(Ref.) | 1(Ref.) | 1(Ref.) |
| 120–139 mg/dL | 137794 | 71003 | 0.91(0.85–0.97) | 1.02(0.97–1.07) | 0.92(0.85–1.00) | 1.01(0.95–1.08) | 0.91(0.87–0.96) | 1.01(0.97–1.05) |
| 140–159 mg/dL | 71035 | 45618 | 1.04(0.97–1.12) | 1.09(1.02–1.15) | 0.98(0.90–1.08) | 1.07(0.99–1.15) | 1.00(0.95–1.05) | 1.03(0.98–1.08) |
| 160–179 mg/dL | 28822 | 25930 | 1.18(1.08–1.28) | 1.28(1.20–1.36) | 1.12(1.01–1.25) | 1.21(1.11–1.31) | 1.14(1.07–1.21) | 1.19(1.13–1.25) |
| ≥180 mg/dL | 43792 | 46364 | 1.59(1.48–1.72) | 1.69(1.60–1.78) | 1.39(1.26–1.52) | 1.45(1.35–1.55) | 1.45(1.38–1.53) | 1.56(1.49–1.62) |
| *P* for interaction |  |  | 0.052 |  | 0.156 |  | <0.001 |  |
| Sulfonylurea |  |  |  |  |  |  |  |  |
| <80 mg/dL | 2745 | 7862 | 1.45(1.22–1.74) | 1.16(1.06–1.28) | 1.43(1.16–1.77) | 1.21(1.07–1.36) | 1.66(1.47–1.87) | 1.37(1.29–1.46) |
| 80–99 mg/dL | 15679 | 34823 | 1.20(1.09–1.33) | 1.07(1.02–1.14) | 1.11(0.98–1.25) | 1.10(1.02–1.18) | 1.29(1.20–1.39) | 1.12 (1.08–1.17) |
| 100–119 mg/dL | 30751 | 62019 | 1(Ref.) | 1(Ref.) | 1(Ref.) | 1(Ref.) | 1(Ref.) | 1(Ref.) |
| 120–139 mg/dL | 139850 | 68947 | 1.02(0.95–1.09) | 1.00(0.96–1.06) | 1.02(0.94–1.11) | 0.99(0.93–1.05) | 1.02(0.97–1.07) | 0.99 (0.96–1.04) |
| 140–159 mg/dL | 69175 | 47478 | 1.18(1.09–1.28) | 1.05(1.00–1.11) | 1.08(0.98–1.19) | 1.04(0.97–1.11) | 1.14(1.07–1.20) | 0.99(0.96–1.04) |
| 160–179 mg/dL | 26468 | 28284 | 1.36(1.24–1.49) | 1.21(1.14–1.29) | 1.26(1.12–1.41) | 1.15(1.06–1.25) | 1.31(1.22–1.40) | 1.14(1.09–1.20) |
| ≥180 mg/dL | 39330 | 50826 | 1.90(1.75–2.06) | 1.58(1.50–1.66) | 1.52(1.37–1.68) | 1.4(1.31–1.49) | 1.65(1.55–1.75) | 1.49(1.43–1.55) |
| *P* for interaction |  |  | <0.001 |  | 0.382 |  | <0.001 |  |
| Meglitinide |  |  |  |  |  |  |  |  |
| <80 mg/dL | 10047 | 560 | 1.22(1.12–1.33) | 1.33(0.95–1.86) | 1.23(1.10–1.37) | 1.83(1.24–2.71) | 1.42(1.34–1.50) | 1.80(1.45–2.24) |
| 80–99 mg/dL | 48663 | 1839 | 1.10(1.05–1.16) | 1.22(0.96–1.54) | 1.09(1.02–1.16) | 1.40(1.05–1.88) | 1.15(1.11–1.19) | 1.54(1.31–1.81) |
| 100–119 mg/dL | 89611 | 3159 | 1(Ref.) | 1(Ref.) | 1(Ref.) | 1(Ref.) | 1(Ref.) | 1(Ref.) |
| 120–139 mg/dL | 205373 | 3424 | 0.96(0.92–0.99) | 1.04(0.84–1.29) | 0.96(0.91–1.01) | 1.06(0.81–1.39) | 0.96(0.93–0.99) | 1.16(0.99–1.35) |
| 140–159 mg/dL | 114294 | 2359 | 1.06(1.01–1.10) | 1.34(1.07–1.67) | 1.01(0.96–1.07) | 1.30(0.98–1.73) | 1.02(0.99–1.05) | 1.18(0.99–1.40) |
| 160–179 mg/dL | 53276 | 1476 | 1.23(1.17–1.30) | 1.25(0.96–1.62) | 1.17(1.09–1.25) | 1.20(0.85–1.68) | 1.16(1.12–1.21) | 1.42(1.17–1.71) |
| ≥180 mg/dL | 87042 | 3114 | 1.65(1.58–1.73) | 1.63(1.33–1.99) | 1.41(1.33–1.49) | 1.76(1.35–2.28) | 1.51(1.46–1.56) | 1.68(1.44–1.95) |
| *P* for interaction |  |  | 0.352 |  | 0.368 |  | 0.016 |  |
| Thiazolidinedione |  |  |  |  |  |  |  |  |
| <80 mg/dL | 9408 | 1199 | 1.22(1.12–1.33) | 1.25(0.95–1.63) | 1.28(1.15–1.42) | 1.10(0.77–1.58) | 1.44(1.36–1.53) | 1.44(1.20–1.74) |
| 80–99 mg/dL | 44283 | 6219 | 1.11(1.05–1.16) | 1.13(0.97–1.32) | 1.08(1.01–1.16) | 1.29(1.07–1.56) | 1.17(1.13–1.22) | 1.11(0.99–1.24) |
| 100–119 mg/dL | 81482 | 11288 | 1(Ref.) | 1(Ref.) | 1(Ref.) | 1(Ref.) | 1(Ref.) | 1(Ref.) |
| 120–139 mg/dL | 197342 | 11455 | 0.94(0.91–0.98) | 1.08(0.95–1.24) | 0.95(0.90–1.00) | 1.09(0.92–1.29) | 0.96(0.93–0.99) | 0.99(0.89–1.09) |
| 140–159 mg/dL | 109177 | 7476 | 1.05(1.00–1.09) | 1.24(1.07–1.43) | 1.00(0.95–1.06) | 1.19(0.99–1.44) | 1.02(0.99–1.06) | 1.08(0.96–1.21) |
| 160–179 mg/dL | 50400 | 4352 | 1.22(1.16–1.29) | 1.30(1.09–1.54) | 1.15(1.07–1.23) | 1.32(1.07–1.64) | 1.17(1.12–1.22) | 1.24(1.09–1.42) |
| ≥180 mg/dL | 81449 | 8707 | 1.63(1.55–1.70) | 1.87(1.63–2.13) | 1.38(1.30–1.46) | 1.83(1.55–2.17) | 1.51(1.45–1.56) | 1.63(1.47–1.81) |
| *P* for interaction |  |  | 0.275 |  | 0.025 |  | 0.772 |  |
| DPP-4 inhibitor |  |  |  |  |  |  |  |  |
| <80 mg/dL | 9820 | 787 | 1.26(1.16–1.37) | 0.71(0.47–1.08) | 1.23(1.10–1.37) | 1.81(1.28–2.56) | 1.43(1.35–1.51) | 1.60(1.29–1.99) |
| 80–99 mg/dL | 45746 | 4756 | 1.10(1.04–1.16) | 1.20(1.01–1.42) | 1.10(1.03–1.18) | 1.08(0.87–1.34) | 1.16(1.11–1.20) | 1.28(1.13–1.46) |
| 100–119 mg/dL | 82257 | 10513 | 1(Ref.) | 1(Ref.) | 1(Ref.) | 1(Ref.) | 1(Ref.) | 1(Ref.) |
| 120–139 mg/dL | 197264 | 11533 | 0.95(0.91–0.99) | 1.08(0.94–1.24) | 0.95(0.90–1.00) | 1.14(0.96–1.35) | 0.96(0.93–0.99) | 1.01(0.91–1.13) |
| 140–159 mg/dL | 109059 | 7594 | 1.05(1.01–1.10) | 1.18(1.01–1.37) | 1.01(0.96–1.07) | 1.14(0.94–1.37) | 1.02(0.99–1.06) | 0.98(0.87–1.11) |
| 160–179 mg/dL | 50361 | 4391 | 1.23(1.16–1.30) | 1.3(1.09–1.55) | 1.15(1.07–1.23) | 1.38(1.11–1.70) | 1.16(1.12–1.21) | 1.29(1.12–1.48) |
| ≥180 mg/dL | 81897 | 8259 | 1.66(1.59–1.74) | 1.57(1.36–1.82) | 1.41(1.33–1.49) | 1.55(1.29–1.84) | 1.52(1.46–1.57) | 1.51(1.35–1.69) |
| *P* for interaction |  |  | 0.008 |  | 0.158 |  | 0.147 |  |
| a-Glucosidase inhibitor |  |  |  |  |  |  |  |  |
| <80 mg/dL | 8267 | 2340 | 1.20(1.09–1.32) | 1.27(1.09–1.50) | 1.18(1.04–1.34) | 1.53(1.26–1.86) | 1.49(1.39–1.59) | 1.29(1.15–1.45) |
| 80–99 mg/dL | 41634 | 8868 | 1.09(1.03–1.16) | 1.15(1.04–1.28) | 1.08(1.01–1.16) | 1.18(1.03–1.36) | 1.17(1.13–1.22) | 1.14(1.05–1.23) |
| 100–119 mg/dL | 77705 | 15065 | 1(Ref.) | 1(Ref.) | 1(Ref.) | 1(Ref.) | 1(Ref.) | 1(Ref.) |
| 120–139 mg/dL | 191628 | 17169 | 0.95(0.91–0.99) | 1.04(0.95–1.14) | 0.94(0.89–0.99) | 1.10(0.97–1.23) | 0.96(0.93–0.99) | 1.02(0.95–1.09) |
| 140–159 mg/dL | 104096 | 12557 | 1.05(1.00–1.11) | 1.14(1.03–1.25) | 0.98(0.93–1.05) | 1.21(1.06–1.37) | 1.03(0.99–1.06) | 1.03(0.96–1.11) |
| 160–179 mg/dL | 46542 | 8210 | 1.23(1.16–1.31) | 1.23(1.10–1.37) | 1.12(1.04–1.21) | 1.33(1.16–1.53) | 1.17(1.12–1.23) | 1.16(1.07–1.26) |
| ≥180 mg/dL | 73485 | 16671 | 1.63(1.55–1.71) | 1.67(1.53–1.82) | 1.35(1.27–1.44) | 1.63(1.45–1.83) | 1.52(1.46–1.57) | 1.47(1.38–1.58) |
| *P* for interaction |  |  | 0.604 |  | 0.045 |  | 0.037 |  |
| Insulin |  |  |  |  |  |  |  |  |
| <80 mg/dL | 8083 | 2524 | 1.17(1.06–1.29) | 1.10(0.94–1.30) | 1.11(0.98–1.26) | 1.26(1.05–1.52) | 1.33(1.24–1.42) | 1.23(1.11–1.37) |
| 80–99 mg/dL | 44344 | 6158 | 1.09(1.03–1.15) | 1.08(0.96–1.23) | 1.08(1.01–1.15) | 1.10(0.95–1.27) | 1.14(1.10–1.19) | 1.10(1.01–1.19) |
| 100–119 mg/dL | 84879 | 7891 | 1(Ref.) | 1(Ref.) | 1(Ref.) | 1(Ref.) | 1(Ref.) | 1(Ref.) |
| 120–139 mg/dL | 200937 | 7860 | 0.96(0.92–1.00) | 1.08(0.96–1.21) | 0.97(0.92–1.02) | 1.00(0.87–1.16) | 0.99(0.96–1.02) | 1.00(0.92–1.08) |
| 140–159 mg/dL | 110965 | 5688 | 1.07(1.02–1.12) | 1.16(1.03–1.32) | 1.02(0.96–1.08) | 1.14(0.98–1.33) | 1.05(1.02–1.09) | 0.99(0.91–1.08) |
| 160–179 mg/dL | 50916 | 3836 | 1.24(1.18–1.31) | 1.21(1.05–1.39) | 1.18(1.10–1.26) | 1.10(0.92–1.31) | 1.19(1.14–1.24) | 1.12(1.02–1.24) |
| ≥180 mg/dL | 80138 | 10018 | 1.64(1.57–1.72) | 1.53(1.37–1.70) | 1.40(1.32–1.49) | 1.31(1.15–1.50) | 1.51(1.45–1.56) | 1.28(1.18–1.38) |
| *P* for interaction |  |  | 0.060 |  | 0.173 |  | 0.007 |  |

**^1^** Adjusted for age, sex, body mass index, alcohol drinking, smoking, regular exercise, presence of hypertension, dyslipidemia, chronic kidney disease, lower 20% income, duration of diabetes over 5 years, and mean of fasting glucose. HR, hazard ratio; CI, confidence interval; ADM, antidiabetic medication.
